# Evaluation of a behavior-centered design strategy for creating demand for oral PrEP among young women in Cape Town, South Africa

**DOI:** 10.12688/gatesopenres.13103.2

**Published:** 2020-07-03

**Authors:** Jennifer F. Morton, Laura Myers, Katherine Gill, Linda-Gail Bekker, Gabrielle Stein, Katherine K. Thomas, Menna Duyver, Ariane van der Straten, Margaret McConnell, Robert Aunger, Valerie Curtis, Jessie de Witt Huberts, Lut Van Damme, Jared M. Baeten, Connie Celum

**Affiliations:** 1 Department of Global Health, University of Washington, Seattle, USA; 2 Desmond Tutu HIV Centre, Cape Town, South Africa; 3 Women's Global Health Imperative, RTI International, San Francisco, USA; 4 Center for AIDS Prevention Studies, Department of Medicine, University of California, San Francisco, USA; 5 Harvard University, Boston, USA; 6 London School of Health and Tropical Medicine, London, UK; 7 Bill & Melinda Gates Foundation, Seattle, USA; 8 Department of Medicine, University of Washington, Seattle, USA; 9 Department of Epidemiology, University of Washington, Seattle, USA

**Keywords:** pre-exposure prophylaxis, young women, HIV prevention, Africa

## Abstract

Background: There is an urgent need to find effective interventions that reduce young South African women’s vulnerability to HIV, and pre-exposure prophylaxis (PrEP) is highly effective when taken consistently. As national programs in Africa launch PrEP programs for young women, it is critical to understand how to effectively create awareness, stimulate interest, and increase uptake of PrEP.

Methods: Behavior-centered design (BCD) guided the development of a PrEP social marketing campaign for young women. Ethnographic observations, in-depth interviews, and focus-group discussions with young South African women informed the content and design of a 90-second PrEP demand creation video and two informational brochures. A short survey was administered to young women at their homes after watching a video to evaluate PrEP interest. Of 800 households with a 16-25-year-old female identified from a Cape Town township census, 320 women in these households viewed the video and completed a survey about the video and their interest in PrEP.

Results: In focus groups, young women from the township preferred local characters and messaging that was empowering, simple, and motivational. From the household survey of young women who viewed the video, most reported interest in learning more about PrEP (67.7% ‘definitely interested’ and 9.4% ‘somewhat interested’) and taking PrEP (56.4% ‘definitely interested’ and 12.5% ‘somewhat interested’). Factors significantly associated with interest in taking PrEP were having a primary partner with whom they regularly have sex (80.0% vs. 65.2% without a primary partner; adjusted odds ratio (AOR)=3.1, 95% CI: 1.3, 7.0) and being in a sexual partnership for <6 months (86.8% vs. 68.5% for >12 months; AOR=3.0, 95% CI: 1.2, 7.3).

Conclusions: A positively framed PrEP demand creation video generated high interest in PrEP among young South African women, particularly among women with a primary partner and a shorter-term relationship.

**Registration:** NCT03142256; registered on 5 May 2017.

## Introduction

Young African women aged 16 to 25 are a priority population for HIV prevention, representing three of the four million young people living with HIV in sub-Saharan Africa, and with high HIV incidence rates of 4–6% in recent HIV prevention trials ^1^. Oral pre-exposure prophylaxis (PrEP) has been proven highly effective as an HIV prevention strategy in clinical trials, demonstration projects and some roll-out settings, with a strong relationship between adherence and effectiveness ^2– 5^. The World Health Organization recommends that PrEP be offered as an HIV prevention option to all people at substantial risk of HIV infection, including adolescent girls and young women (AGYW) ^6^. Given low levels of awareness, effective strategies to stimulate interest in and demand for this new prevention method are needed as South Africa begins providing PrEP to AGYW.

Behavior-centered design (BCD) is a framework for designing behavior change interventions ^7^. In BCD, changes in behavior are viewed as the consequence of a reinforcement learning process. Rather than focus on health messaging, BCD interventions aim to use emotional levers or environmental modifications to create demand and change behavior. Within public health, BCD has informed the design of a number of effective hygiene and sanitation interventions ^7– 15^. The BCD process has five steps to behavior change: ‘Assess’, ‘Build’, ‘Create’, ‘Deliver’, and ‘Evaluate’.

## Methods

The 3Ps (Perception, Partners, Pills) for Prevention Study is a PrEP demonstration project that evaluated uptake and adherence to PrEP among young women in a township of approximately 25,000 people near Cape Town, South Africa. From 2015 to 2017, a PrEP demand creation campaign included the following phases.

### Phase I (Assess): Gather existing knowledge and develop a working hypothesis

The first step of the BCD process involves assessing what is known about the target audience (South African AGYW), the behavior of interest (PrEP uptake and adherence), and the parameters of the intervention in order to establish a hypothesis about how behavior change may be achieved. A ‘framing workshop’ was convened with 28 key stakeholders, including community outreach and community advisory board members, socio-behavioral scientists and qualitative interviewers, and young women who had participated in a small research study of PrEP ^16^ to review available evidence, map young women’s daily activities to identify points of contact, and identify social networks and influencers that could be leveraged to increase young women’s interest in and uptake of PrEP.

### Phase II (Build): Formative research with the target audience

The second BCD step involves carrying out formative research to gather additional information and explore hypotheses about likely drivers of change. We conducted interviews with 37 participants from the target community to generate insight into possible motivations for HIV prevention, including use of PrEP. Participants included 11 PrEP-naïve young women (aged 16–25), 10 PrEP-experienced women (aged 16–29), five older women living with HIV (aged 26–32), four men (aged 25–35), and seven key informants (two clinic workers, a teacher, social worker, politician, community-based organization worker, and shop owner). Selection criteria included age, gender, location, previous PrEP experience (young women), HIV status (older women), and leadership role in the community (key informants); efforts were made to include an equal number of participants from formal and informal settlements within the research community. PrEP-naïve young women were recruited using a snowball method during the first week, which yielded participants who were familiar with the research organization. In order to reach a more representative sample, additional participants were randomly sampled using household data from a census in the township which the research team conducted during the previous year. PrEP-experienced young women were recruited from prior PrEP research studies. Women living with HIV, men, and key informants were purposefully recruited by community outreach workers. Study staff phoned or visited potential participants, explained what participation involved and arranged an interview time for those willing to participate. Respondents were people who matched the selection criteria and from whom we got written consent (or assent with parental consent if they were younger than 18).

Ethnographic interviews with young women lasted between two and three hours and sampled from 20 participatory activities (including motivational and social network mapping, product attribute ranking, word association, daily scripting, and household inventories) to better understand young women’s daily routines, hopes, concerns, social life, relationships, health-seeking behavior, perceptions of HIV prevention and PrEP, and potential points of contact to learn about PrEP. Follow-up interviews were carried out with four participants who consented to participate in an optional photovoice activity. In-depth interviews with women living with HIV, men, and key informants lasted 60–90 minutes and explored general awareness of PrEP, drivers of HIV, community expectations of young women, and household configurations (relevant to young women’s ability to discretely store PrEP pill bottles). Interviews were conducted at participants’ homes or workplaces by experienced behavioral science researchers with graduate training and no prior relationship to participants from the London School of Hygeine and Tropical Medicine (RA, JdWH, VC) and the Desmond Tutu HIV Centre (LM). A female community outreach worker (TM) who lived in the research community and was acquainted with some participants provided translation, when needed. Interviews were audio-recorded and case profiles were written for each household based on interview and process notes; demographic information and responses to specific BCD activities were captured in an Excel spreadsheet.

Data was analyzed by ‘sequential recycling’, a consumer research process designed to explore all directions that might lead to an effective intervention by closing down avenues of investigation when they appear exhausted or irrelevant and moving into new areas of investigation when they arise. Interviewers noted significant or surprising anecdotes, patterns, and associations during fieldwork brainstorming sessions in the case reports. To ensure behavioral determinants were sufficiently researched, two researchers (RA, JdWH) sorted items into a ‘BCD checklist’ and either classified them as findings (when there was agreement), questions for further exploration, or set them aside as irrelevant; underrepresented topic areas were marked for further investigation. When the fieldwork was completed, they combed the data for reoccurring themes, patterns, and associations, which were clustered into causal categories according to the BCD checklist, from which specific insights and prototype messages to leverage interest in PrEP emerged. These were taken into the field for hypotheses testing in a second stage of formative interviews with nine PrEP-naïve young women (aged 19–26), one PrEP-experienced young woman (aged 17), 11 men (aged 22–33), and one woman living with HIV (aged 29).

Themes and insights generated from this formative research and previous qualitative work about HIV prevention and PrEP in this community ^17^ were presented at a two-day creative workshop involving community outreach staff, youth interns, community advisory board members, young women from the community, investigators, representatives from a marketing firm, and research staff. A participatory process tested the resonance of the formative findings, narrowing 40 themes down to five overarching insights and further reduced to a single insight, from which a BCD theory of change was developed as the foundation for a creative process to promote PrEP among South African AGYW. To check for resonance, the themes and overarching insights were then pilot-tested with nine PrEP-experienced AGYW aged 16–19 that were recruited from another PrEP study. The two-hour discussion was facilitated by a female Behavioral Scientist (LM) and a male isiXhosa-speaking Social Science Interviewer (NSM) who did not have a prior relationship with participants. The guide produced for the discussions is available as *Extended data* ^18^.

### Phase III (Create): Develop and test intervention package

The third BCD phase involves iteratively designing and testing communication materials. The marketing firm was given a creative brief and developed seven initial PrEP demand creation campaign concepts, of which the research team selected four. These visual and slogan-based materials were then presented for discussion and ranking by four groups of young women aged 17–24 from the community (n=38) to identify which elements young women liked and resonated with the most. Study staff purposefully recruited (face-to-face and telephonically) three groups of PrEP-naïve women and one with participants from another PrEP study. Participants were selected according to age (16–24 years), gender (female), residence in the study community, and PrEP experience. Interested participants were invited to a two to three-hour session at the research office in which a female Behavioral Scientist (LM) and male isiXhosa-speaking Social Science Interviewer (NSM) who did not have a prior relationship with participants facilitated the discussion and took detailed notes, from which a comprehensive summary report was written. A final concept and video script were developed and revised with feedback from additional youth and the research team. A professional filmmaker cast local youth and filmed a 90-second video (available as *Extended data* ^18^) in the community.

### Phase IV (Deliver) and V (Evaluate): Deliver and evaluate a motivating intervention

The fourth and fifth steps of the BCD process involve delivering an intervention using appropriate channels and evaluating it along the theory of change. To determine the effect of the PrEP video on young women’s interest in PrEP, 800 households with a young woman between ages of 16 and 25 were randomly selected using a community census conducted the previous year. Between March and October 2017, research staff visited each of the randomly selected houses up to three times, requesting to speak with the young woman household resident. Young women who agreed to participate were shown the video and then asked to self-administer a short survey that included questions on demographics, sexual relationships, risk-taking, HIV risk perception, PrEP interest and knowledge, and their opinions about the video. A copy of the survey is available as *Extended data* ^18^. All questions had been used in previous research except those specific to participant’s opinions about the video. Women who reported interest in learning more about PrEP were referred to the research clinic. In addition, 18 survey participants who had seen the video during a household visit (13 of whom subsequently initiated PrEP and five who opted not to initiate PrEP) took part in two FGDs. A semi-structured discussion guide elicited feedback on the video and brochures. FGDs lasted between 1.5 and 2 hours and were held at the research office, facilitated by a female Behavioral Scientist (LM), translated by a female Social Science Interviewer (NM), audio-recorded, and transcribed ^18^. The focus group discussion guides are available as Extended data. The de-identified transcripts are available as Underlying data.

### Statistical analysis

Characteristics of young women were summarized using descriptive statistics. Interest in taking PrEP was defined as being either ‘definitely’ or ‘somewhat’ interested vs. ’unsure’, or ‘definitely’ or ‘somewhat’ uninterested. Associations between characteristics and the outcome of interest in taking PrEP were estimated as odds ratios (ORs) from logistic regression. Characteristics considered for association were: age, education, food security, wanting a child in the next year, having a primary sexual partner, partner age difference >5 years, partnership duration, sex with primary partner in prior 30 days, sex with anyone else, risk taking, perception of participant’s own HIV risk, whether participant had heard of PrEP prior to the survey, how they had heard of PrEP, and whether the participant liked the video. P-values were from likelihood ratio tests. Adjusted odds ratios (AORs) were generated using multivariate logistic regression including all characteristics with p<0.20 in unadjusted analysis. The statistical testing significance level was α=0.05. All analyses were conducted using SAS version 9.4 (SAS Institute). 

### Ethical approval

The study was reviewed and approved by the University of Cape Town’s Health Research Ethics Committee (ref 567/2016). Informed consent was obtained for the interviews, FGDs and surveys.

## Results

### Phases I (Assess) and II (Build): Formative research and message development

Ethnographic research identified multiple barriers to young women’s interest in PrEP, including low concern about HIV, low sense of personal risk of HIV acquisition, cultural norms in which preventative pill-taking is unusual, concern about possible side effects, and socio-economic stressors that reduce personal agency and future-oriented thinking. Findings suggested that a successful demand creation strategy could harness young women’s desire to increase their attractiveness to partners, increase emotional intimacy, avoid the social consequences of HIV, and become role models through becoming early adopters of PrEP.

Final themes to make PrEP a desirable prevention strategy from the creative workshop included:

1) PrEP enhances the power you have (‘You call the shots, make your own decisions, are independent and don’t have to rely on your partner to prevent HIV’)
2) Take care of yourself; PrEP increases your self-worth (‘Taking the pill gives you the opportunity to be part of an ‘exclusive group’ that is turning the tide against HIV’)
3) PrEP protects those you love (‘Women who don’t get HIV protect their family from shame, financial burden and grief’)
4) PrEP allows you to live in the sexual moment (‘PrEP helps you stay in the mood and enhances sexual pleasure and intimacy’)
5) PrEP helps children (‘Mothers can die from HIV, leaving others with the burden of raising their children’)

Young women endorsed the first two themes which affirmed their agency and capacity to make decisions. With that feedback, the marketing agency proposed the central insight ‘PrEP is about me and not the next person. It’s my life, it’s my future, it’s my decision’ and developed four related concepts. In FGDs, the majority of young women favored the concepts that emphasized how PrEP facilitates young women’s control of their lives: ‘I am PrEPPed’ and ‘We are the generation to end HIV’. One participant said, *“I like the fact that they have big dreams for themselves”* (PrEP-naive female). De-identified transcripts of FGDs are available as *Underlying data* ^18^.

### Phase III (Create): Develop and test demand creation materials

During testing, young women preferred clear messaging about what PrEP is and wanted that messaging to be empowering, simple, and motivational. While young women preferred realistic visuals set in the township, they also wanted materials to feel aspirational, with images of young women who looked similar to them, rather than highly stylized models. Some young women thought the campaign should also include young men; for example, two participants said this would demonstrate that PrEP “ *is for everyone*” (PrEP-naive female) and “ *girls will not have to do this fight alone*” (PrEP-naive female). Young women also found the prospect of being part of *‘the generation to end HIV’* appealing:

*“These big words—we are the generation that will end HIV, it’s like it brings light, like there is something that can put an end to HIV… It is up to each of us to do our part…You don’t have to depend on someone else, you can do it for yourself”* (PrEP-naïve female).

### Phase IV (Deliver) and V (Evaluate): Deliver and evaluate the PrEP demand creation video

The final demand creation materials included a 90-second PrEP video and two brochures—one to provide more detailed information about PrEP to young women and one to help young women introduce PrEP to their friends, parents, and partners (see *Extended data*) ^18^. Participants in FGDs who were shown the final PrEP video described the video as ‘encouraging’, ‘motivating’, ‘exciting’, ‘mind-opening’, and ‘informative’. Characters were perceived positively, as taking care of themselves by taking PrEP. One participant recalled *“when I saw that video, seeing people who were in there, I became interested. They were proud, energetic” (F1, PrEP acceptor).* Participants liked that the group of friends supported and encouraged each other and appreciated the inclusion of male characters, which demonstrated that *“guys can also take part; it’s not only the girls” (F5, PrEP decliner).* The character publicly swallowing her PrEP pill highlighted to many that PrEP is easy to take and does not need to be hidden from others: *“I liked the part where that girl took the pill. It shows that it’s not hard to take the pill every day” (F1, PrEP decliner).* Some participants suggested they would not have been as motivated to try PrEP without the video, which provided credibility to the idea of a pill preventing HIV, for example: *“no one would have believed [if we weren’t] shown a video… How would we know if [the recruiter] is telling the truth or if she is lying?” (F10, PrEP acceptor).* A participant who saw the PrEP video at home recalled her excitement— *"I didn’t follow up, but I was excited at the moment, that I actually really want to do this” (F4, PrEP decliner)*. Suggested improvements for the video included more character dialogue, detailed information about PrEP, a PrEP-user sharing her own experience, and being broadcast by television or radio.

Household survey about PrEP interest among young women:

Of 800 households randomly selected from the community census, study staff confirmed a young woman aged 16–25 lived in 497 households. Of those, 320 (64.4%) consented to see the video and complete a short survey, 83 (16.7%) refused to participate, and 94 (18.9%) were not home during any of three visits to their household Table 1. The raw results of the survey are available as *Underlying data* ^18^.

**Table 1.** Participant characteristics among survey population.

Characteristic	Value, n (%) or median (IQR)
Households surveyed	497
Consented Participants	320 (64.4%)
*Demographics and Partner Questions*	
Age in years	20 (18, 23) *
Education	
Any primary school	70 (21.9%)
Any secondary school	215 (67.2%)
Any tertiary school	32 (10.0%)
Prefer not to answer	2 (0.6%)
Food Security in the Past 30 Days	
Never worried	101 (31.6%)
Sometimes worried	166 (51.9%)
Often worried	37 (11.6%)
Prefer not to answer	15 (4.7%)
Wants child in the next year	56 (17.5%)
Primary sexual partner	173 (54.1%)
Age of primary sexual partner (years)	25 (22, 27)
Age of primary sexual partner is at least 5 years older than participant	60 (18.6%)
Partnership duration in months	8 (4, 24) *
Sex with primary partner (last 30 days)	131 (40.9%)
Sex with anyone else (last 30 days)	59 (18.4%)

*Median (IQR).

Most women reported positive feelings about the video and were interested in PrEP after viewing it; 70.8% rated the video between 7–10 on a scale from 0–10 (0 indicated they really disliked the video, five that the video was okay, and 10 that they really liked video). Overall, 58.8% had ‘mostly positive feelings’ watching the video, 60.2% related to the characters ‘a lot’, and 78.7% would recommend the video to a friend. The majority of women were interested in learning more about PrEP (67.7% ‘definitely interested’ and 9.4% ‘somewhat interested’) and taking PrEP (56.4% ‘definitely interested’ and 12.5% ‘somewhat interested’). Among women who did not report being ‘definitely interested’ in taking PrEP, 48.3% reported that ‘learning more about PrEP’ would make them more interested. A minority (10.8%) reported having a friend or family member taking PrEP would make them more interested and 8.3% reported they would be more interested in taking PrEP if a healthcare worker recommended it to them.

In multivariate analysis, interest in taking PrEP was associated with having a primary partner with whom they regularly have sex (132/165 [80.0%] vs. no primary partner 88/135 [65.2%], AOR =3.1; 95% CI=1.3, 7.0) and with being in a shorter relationship (<6 months (66/76 [86.8%] vs. >12 months 37/54 [68.5%], AOR=3.0; 95% CI=1.2, 7.3; Table 2).

**Table 2.** Associations with interest in taking pre-exposure prophylaxis (PrEP).

Characteristic ^1^:	Value, n	Interested in taking PrEP, n (%)	Odds ratio (95% CI)	p-value	Adjusted odds ratio ^2^ (95% CI)	p-value
Consented participants with outcome measured	300	220 (73.3%)				
Has primary sexual partner	165	132 (80.0%)	2.1 (1.3,3.6)	0.004	3.1 (1.3, 7.0)	0.005
Age of primary sexual partner is at least 5 years older than participant	55	46 (83.6%)	2.1 (1.0, 4.5)	0.05	1.3 (0.5, 3.2)	0.54
Partnership duration (months)				0.002		0.04
0–6 Months	76	66 (86.8%)	3.0 (1.3, 7.3)		3.0 (1.2, 7.3)	
6–12 Months	35	29 (82.9%)	2.2 (0.8, 6.3)		2.4 (0.8, 7.2)	
> 12 Months	54	37 (68.5%)	*reference*		*reference*	

^1^Characteristics considered were: age, education, food security, wanting child in next year, primary sexual partner, age difference with partner >5 years, partnership duration, sex with primary partner in prior 30 days, sex with anyone else, risk taking, perception of participant’s own HIV risk, whether participant had heard of PrEP prior to the survey, how they had heard of PrEP, and whether the participant liked the video. ^2^Multivariate logistic regression models were adjusted for variables shown in the table as well as food security (p=0.13 and 0.19, in univariate and multivariate models, respectively) and risk taking (p=0.20 and 0.12).

## Discussion

PrEP demand creation materials for young South African women were developed using a behavior-centered design framework, ethnographic and qualitative research, and an iterative process. Members of the target audience (young women residing in a township), community members, a marketing firm, and a research team experienced in PrEP delivery reviewed the materials during each iteration. Initial formative research identified a primary communication challenge: convincing healthy young women with low motivation to take a pill to prevent HIV, in part due to the burden of taking a pill daily with potential side effects to prevent a disease for which they do not currently or consistently feel at risk. Key messages and themes generated by researchers and a marketing firm needed to be refined several times during the BCD process before being enthusiastically endorsed by young women. Young women rejected messaging that emphasized risk behavior and images that did not feel authentic, preferring characters and scenes from their local community and aspirational messaging that emphasized their control over their lives and health.

A 90-second video, available as *Extended data* ^18^, was developed to stimulate interest in PrEP that featured positive framing and relatable images of young women in the township. Over two-thirds of young women reported interest in PrEP after viewing the video, with greater interest in PrEP among women with a primary partner and a shorter duration of partnership. The materials were tailored to the township in which the study occurred but have been used by other PrEP studies and demonstration projects in Kenya and South Africa and could be further disseminated to other countries that want to raise awareness and generate demand for PrEP among young African women.

HIV prevention communication should be positive and engaging and reflect the community and population for which it will be delivered. Iterative feedback from the target audience should be sought to refine the concept, images and messaging. Although these demand creation materials were tested with young women and the majority reported liking the video, given low general public awareness about PrEP in many African settings, it is likely that young women may need to encounter PrEP messaging multiple times and through multiple channels before actively seeking PrEP. We expect that social marketing campaigns that additionally target others who influence young women (friends, partners, family) will have a positive effect on uptake by creating more supportive behavior settings in which young women initiate PrEP.

This study has a number of limitations. Most of the formative work conducted to develop the content and esthetic of the video was conducted by researchers who are not part of the local community and who do not speak the local language; this influence may have made themes considered for the video less reflective of the community. However, many efforts were made throughout the ethnographic research and development of the video to check in with local community and in particular young women to test ideas and concepts prior to adoption. Additionally, a major limitation of this study is use of ‘interest in PrEP’ as a proxy for if the materials would influence a young woman to seek out PrEP, without a reliable measure of subsequent initiation of PrEP among surveyed participants. Lastly, during the time of this PrEP demand creation campaign, South African PrEP awareness campaigns were launched to which participants may have had some exposure.

In summary, PrEP is a highly effective but novel HIV prevention method for which strategies that build awareness and generate demand for PrEP across multiple communication channels are needed. We expect that PrEP communication materials that capture viewers’ attention, introduce how this new prevention strategy works, and convey powerfully motivating reasons for trying it will drive many young women to learn more about PrEP. Behavior-centered design offers a useful framework with which to develop PrEP demand creation strategies and materials.

## Data availability

### Underlying data

Harvard Dataverse: 3P Demand Creation. https://doi.org/10.7910/DVN/RIR3UP ^18^.

This project contains the following underlying data:

- 3P_Demand Creation_FGD1_Transcript.pdf (transcript for focus group discussion 1).
- Creation_FGD2_Transcript.pdf (transcript for focus group discussion 2).
- 3P_DemandCreation.tab (underlying data for video survey).

The Behavior Centered Design ethnographic observations and in-depth interviews were collected at a household level in a small township and contain information that could not be sufficiently de-identified. To preserve participant confidentiality, these data have not been made publicly available. The Principal Investigators of this study, Connie Celum ( ccelum@uw.edu) and Linda-Gail Bekker ( Linda-Gail.Bekker@hiv-research.org.za), may be contacted with requests to access these data. Access may be granted to those wishing to use the data for research purposes.

### Extended data

Harvard Dataverse: 3P Demand Creation. https://doi.org/10.7910/DVN/RIR3UP ^18^.

This project contains the following underlying data:

- 3P_Demand Creation_BCD interview guide.pdf (Interview guide)
- 3P_Demand Creation_Brochure 1.pdf (participant information brochure 1).
- 3P_Demand Creation_Brochure 2.pdf (participant information brochure 2).
- 3P_Demand Creation_Code.sas (analysis code).
- 3P_Demand Creation_DataDictionary.tab (data dictionary for video survey).
- 3P_Demand Creation_FGD guide.pdf (guide for focus group discussions).
- 3P_Demand Creation_PrEP Video.mp4 (PrEP demand creation video).
- 3P_Demand Creation_Video Survey.docx (blank copy of video survey).

Data are available under the terms of the Creative Commons Zero “No rights reserved” data waiver (CC0 1.0 Public domain dedication).

## References

[1] Evidence for Contraceptive Options and HIV Outcomes (ECHO) Trial Consortium: HIV incidence among women using intramuscular depot medroxyprogesterone acetate, a copper intrauterine device, or a levonorgestrel implant for contraception: a randomised, multicentre, open-label trial. *Lancet.* 2019;394(10195):303–13. 10.1016/S0140-6736(19)31288-7 31204114 PMC6675739

[2] Grant RM Lama JR Anderson PL: Preexposure chemoprophylaxis for HIV prevention in men who have sex with men. *N Engl J Med.* 2010;363(27):2587–99. 10.1056/NEJMoa1011205 21091279 PMC3079639

[3] Baeten JM Donnell D Ndase P: Antiretroviral prophylaxis for HIV prevention in heterosexual men and women. *N Engl J Med.* 2012;367(5):399–410. 10.1056/NEJMoa1108524 22784037 PMC3770474

[4] Choopanya K Martin M Suntharasamai P: Antiretroviral prophylaxis for HIV infection in injecting drug users in Bangkok, Thailand (the Bangkok Tenofovir Study): a randomised, double-blind, placebo-controlled phase 3 trial. *Lancet.* 2013;381(9883):2083–90. 10.1016/S0140-6736(13)61127-7 23769234

[5] Thigpen MC Kebaabetswe PM Paxton LA: Antiretroviral preexposure prophylaxis for heterosexual HIV transmission in Botswana. *N Engl J Med.* 2012;367(5):423–34. 10.1056/NEJMoa1110711 22784038

[6] WHO: Consolidated guidelines on the use of antiretroviral drugs for treating and preventing HIV infection. Geneva, Switzerland,2016 Reference Source 27466667

[7] Aunger R Curtis V: Behaviour Centred Design: towards an applied science of behaviour change. *Health Psychol Rev.* 2016;10(4):425–46. 10.1080/17437199.2016.1219673 27535821 PMC5214166

[8] Curtis V Aunger R: Motivational mismatch: Evolved motives as the source of – and solution to – global public health problems. *Applied Evolutionary Psychology.*Oxford: Oxford University Press;2011;259–75. 10.1093/acprof:oso/9780199586073.003.0016

[9] Czerniewska A Muangi WC Aunger R: Theory-driven formative research to inform the design of a national sanitation campaign in Tanzania. *PLoS One.* 2019;14(8):e0221445. 10.1371/journal.pone.0221445 31442255 PMC6707585

[10] Curtis V Dreibelbis R Buxton H: Behaviour settings theory applied to domestic water use in Nigeria: A new conceptual tool for the study of routine behaviour. *Soc Sci Med.* 2019;235:112398. 10.1016/j.socscimed.2019.112398 31326766

[11] Tidwell JB Chipungu J Bosomprah S: Effect of a behaviour change intervention on the quality of peri-urban sanitation in Lusaka, Zambia: a randomised controlled trial. *Lancet Planet Health.* 2019;3(4):e187–e96. 10.1016/S2542-5196(19)30036-1 31029230

[12] Biran A Schmidt WP Varadharajan KS: Effect of a behaviour-change intervention on handwashing with soap in India (SuperAmma): a cluster-randomised trial. *Lancet Glob Health.* 2014;2(3):e145–e54. 10.1016/S2214-109X(13)70160-8 25102847

[13] Greenland K Chipungu J Curtis V: Multiple behaviour change intervention for diarrhoea control in Lusaka, Zambia: a cluster randomised trial. *Lancet Glob Health.* 2016;4(12):e966–e77. 10.1016/S2214-109X(16)30262-5 27855872

[14] Gautam OP Schmidt WP Cairncross S: Trial of a Novel Intervention to Improve Multiple Food Hygiene Behaviors in Nepal. *Am J Trop Med Hyg.* 2017;96(6):1415–26. 10.4269/ajtmh.16-0526 28719285 PMC5462581

[15] Watson J Dreibelbis R Aunger R: Child's play: Harnessing play and curiosity motives to improve child handwashing in a humanitarian setting. *Int J Hyg Environ Health.* 2019;222(2):177–82. 10.1016/j.ijheh.2018.09.002 30219482

[16] Gill K Dietrich J Gray G: Pluspills: an open label, safety and feasibility study of oral pre-exposure prophylaxis (PrEP) in 15-19 year old adolescents in two sites in South Africa. *9th IAS Conference on HIV Science;*July 23–26; Paris, France2017 Reference Source

[17] Hartmann M McConnell M Bekker LG: Motivated Reasoning and HIV Risk? Views on Relationships, Trust, and Risk from Young Women in Cape Town, South Africa, and Implications for Oral PrEP. *AIDS Behav.* 2018;22(11):3468–3479. 10.1007/s10461-018-2044-2 29404757 PMC6077112

[18] Celum C: 3P Demand Creation. Harvard Dataverse, V2,2020 10.7910/DVN/RIR3UP

