# Supplementary material for: Evaluation of a behavior-centered design strategy for creating demand for oral PrEP among young women in Cape Town, South Africa
Source: Gates Open Res. 2020 Jul 3;4:29. Originally published 2020 Mar 10. [Version 2] doi: 10.12688/gatesopenres.13103.2 (PMC7196718; doi:10.12688/gatesopenres.13103.2)
Supplement: Peer review history

# Peer review: Reviewer response for version 1

**DOI:** 10.21956/gatesopenres.14266.r28676

**Published:** 2020-05-01

**Authors:** Saiqa Mulick

**Affiliations:** 1 Wits Reproductive Health Institute, University of the Witwatersrand, Johannesburg, South Africa

Understanding how best to create demand for new HIV prevention technologies is an essential component of normalizing HIV prevention and stimulating uptake of these technologies at scale. This paper clearly describes a systemmatic process to develop a demand creation video and brochures for a PrEP delivery project serving adolescent girls and young women, a high priority target group for HIV prevention technologies in South Africa.

 Although the paper is an excellent contribution to the literature on demand creation for PrEP it can be further strengthened if the authors consider including more detail on the following:

1. How social desirability response bias was minimized during the evaluation of the video
2. Young men were part of the process and were prominent in the video - are there data on whether it may have also stimulated interest in PrEP amongst young men.
3. What participants disliked in previous iterations of the video, messages or imagery (ie what not to do).
4. There is not much information on the brochures. Was there any feedback from survey participants on the brochures and did some women prefer them to the video.

Is the work clearly and accurately presented and does it cite the current literature?

Yes

If applicable, is the statistical analysis and its interpretation appropriate?

Yes

Are all the source data underlying the results available to ensure full reproducibility?

Yes

Is the study design appropriate and is the work technically sound?

Yes

Are the conclusions drawn adequately supported by the results?

Yes

Are sufficient details of methods and analysis provided to allow replication by others?

Yes

Reviewer Expertise:

Implementation Science, PrEP delivery, integration of HIV and SRH, adolescent girls and young women, school-based health, Sexually Transmitted infections, contraception and family planning

I confirm that I have read this submission and believe that I have an appropriate level of expertise to confirm that it is of an acceptable scientific standard.

# Response

**Published:** 2020-06-05

**Authors:** Connie Celum

The study team would like to thank Dr. Mullick for the careful review. Our responses are below:

**1. How social desirability response bias was minimized during the evaluation of the video.**

*Young women were shown the video on a tablet that a study staff person carried with them to the houses which were selected from the census based on a young woman resident and randomized to watch the video. After watching the video on the tablet, the survey was opened and the tablet handed back to the participant so she could complete the survey. Participants could ask the staff person for help. In this situation, the participant submitted the survey on her own. We have added text to the methods section to reflect this.*

**

**2. Young men were part of the process and were prominent in the video - are there data on whether it may have also stimulated interest in PrEP amongst young men?**

*Thank you for this question. Unfortunately, studying men and their interest in PrEP was not formally ascertained in this study;  the video was not pilot tested with men and they were not shown the video nor were they surveyed by study staff. Men, however, were able to access the video through some of the wider demand creation activities and could learn more about PrEP through a youth center connected with the research center but we do not have data about this.*

**

**3. What participants disliked in previous iterations of the video, messages or imagery (ie what not to do).**

*Descriptions of what young women liked and did not like about early versions of the demand ******creation materials are included in the Results section under Phase III (Create): Develop and test demand creation materials.*

*Feedback from young women about early versions of the demand creation campaign included less acceptability for affluent-appearing women in the video and brochures. Young women expressed wanting to see a campaign that featured women like them from the township, but that also had inspirational messages and imagery.   Early versions also only had images of women; young women reported that men are important to young women’s motivation to take PrEP and thus an important secondary audience. Young women also felt that early versions of the materials that talked about PrEP as a ‘new kind of HIV prevention’ did not have enough information about how PrEP is used; we addressed this in the video by explicitly explaining the PrEP is a pill you take once a day and likened it to birth control.*

**

**4. There is not much information on the brochures. Was there any feedback from survey participants on the brochures and did some women prefer them to the video?**

*While we captured young women’s opinions about the content and target audience for the brochures before finalizing them, the content and look of the brochures and preferences for the video or brochures were not formally evaluated in the survey.*

# Peer review: Reviewer response for version 1

**DOI:** 10.21956/gatesopenres.14266.r28675

**Published:** 2020-04-27

**Authors:** Maryam Shahmanesh

**Affiliations:** 1 Institute for Global Health, University College London, London, UK

HIV prevention particularly amongst adolescent girls and young women in South Africa remains a key global health priority. Antiretroviral based pre- exposure prophylaxis is highly effective in reducing the risk of HIV acquisition and has been hoped to be a game-changer when used in combination with other HIV prevention methods. However, effective use will require uptake by those who need (and would benefit from it most), and as a daily tablet during seasons of risk, effective use once taken up. There is therefore an urgent need to increase awareness, stimulate interest and motivate uptake of PrEP as part of HIV prevention. 

 It was really refreshing to see a theoretically informed behaviour change intervention development clearly laid out, with all the steps defined and described. I was particularly impressed to see the iterative and participatory development of the intervention clearly laid out in series of steps to create an intervention which was then evaluated for feasibility and acceptability with the target audience.  

 It was interesting to note that this painstaking and methodological approach to participatory intervention development produced outputs that may not have been intuitive to public health messaging. ie. rather than talk of risk, prevention, or even healthiness - the focus that emerged from the data was motivational messaging that placed the young women at the vanguard of creating and AIDS free generation. The importance of including young men alongside the women, which resonates with other work that is emerging, is also refreshing, as was the need to see the women taking the pill.

 Areas that may have strengthened the paper:

1. I wondered why so much of the formative work was conducted by non isiXhosa speaking social scientist interviewers. There seems to have only been one (NSM). How did this effect the outcomes of the study? I would have liked to have seen some reflection on this "otherness" on the outcomes in the results.  
2. I would have liked to see how many of the young women who received the video actually attended one of the PrEP clinics to discuss suitability.

Is the work clearly and accurately presented and does it cite the current literature?

Yes

If applicable, is the statistical analysis and its interpretation appropriate?

Yes

Are all the source data underlying the results available to ensure full reproducibility?

Yes

Is the study design appropriate and is the work technically sound?

Yes

Are the conclusions drawn adequately supported by the results?

Yes

Are sufficient details of methods and analysis provided to allow replication by others?

Yes

Reviewer Expertise:

HIV prevention; Infectious disease epidemiology; complex intervention development and evaluation; and implementation science.

I confirm that I have read this submission and believe that I have an appropriate level of expertise to confirm that it is of an acceptable scientific standard.

# Response

**Published:** 2020-06-05

**Authors:** Connie Celum

The study team would like to thank Dr. Shamaneh for the careful review. Our responses to the review are below:

**1. I wondered why so much of the formative work was conducted by non isiXhosa speaking social scientist interviewers. There seems to have only been one (NSM). How did this effect the outcomes of the study? I would have liked to have seen some reflection on this "otherness" on the outcomes in the results.**

*The ethnographic interviews conducted during the formative work using Behavior Centered Design (BCD) are a specific and dynamic type of interview that takes extensive training. Unfortunately, due to time constraints the BCD team could not train the site Xhosa speaking interviewers on the ethnographic interviewing. As a contingent plan, we paired each of the trained interviewers with a female community outreach worker who was Xhosa-speaking, lived in the community, and was acquainted with participants.* *The purpose of the formative work was to inform the research team’s understanding of the challenges faced by young women in the community and messages that would resonate with them. To ensure that the information gathered in the formative work was reflective of the community, themes were reviewed by staff and other representatives from the community who attended the creative workshop. Once the themes had been validated by the community members at the creative workshop, we conducted focus group discussions with additional young women from the community to test the messages that had been developed.*

*These focus group discussions were co-facilitated by one of the researchers (LM) who had experience in media development and one of the isiXhosa speaking social science interviewers (NSM). These two co-facilitated the FGDs as they brought unique perspectives (from the video development side and from the community perspective side) to the discussion.*

*The video was developed using an iterative process that repeatedly sought community input and review. After each iteration of feedback from the community, the visuals and content were updated to ensure that the video was reflective of the community and met the needs of the target audience. We also used a random sample of young women from the community to evaluate the video.*

*We recognize that having non-isiXhosa speaking researchers conducting the interviews and focus group discussion may have impacted the development of the video and have included this as a limitation in the manuscript.*

**2. I would have liked to see how many of the young women who received the video actually attended one of the PrEP clinics to discuss suitability.**

*We do not have data on the proportion of young women who saw the video subsequently attended one of the PrEP educational events because locator information was not collected through social media such as Facebook, and unique IDs were not assigned at the educational events in order to link to the household survey. However, of the 320 women who saw the video at home and completed a survey, 35 (10.9%) were enrolled into the 3Ps for Prevention cohort study, which tested the effect of an incentive conditioned on high intracellular tenofovir diphosphate (TVF-DP) levels in dried blood spots at 1, 2, and 3 months. It is difficult to precisely quantify the proportion of women in the community who viewed the video through FaceBook, friends sharing it, or through community events, all of which contributed to recruitment into the 3P cohort study. Furthermore, women who were interested in taking PrEP through the 3Ps for Prevention cohort had to meet eligibility criteria for and be interested in participating in a randomized study.*
